# Global research trends of artificial intelligence applied in esophageal carcinoma: A bibliometric analysis (2000-2022) *via* CiteSpace and VOSviewer

**DOI:** 10.3389/fonc.2022.972357

**Published:** 2022-08-25

**Authors:** Jia-xin Tu, Xue-ting Lin, Hui-qing Ye, Shan-lan Yang, Li-fang Deng, Ruo-ling Zhu, Lei Wu, Xiao-qiang Zhang

**Affiliations:** ^1^ School of Public Health, Nanchang University, Nanchang, China; ^2^ Jiangxi Provincial Key Laboratory of Preventive Medicine, Nanchang University, Nanchang, China; ^3^ Department of Thoracic Surgery, The Second Affiliated Hospital of Nanchang University, Nanchang, China

**Keywords:** esophageal cancer, artificial intelligence, bibliometric, CiteSpace, VOSviewer

## Abstract

**Objective:**

Using visual bibliometric analysis, the application and development of artificial intelligence in clinical esophageal cancer are summarized, and the research progress, hotspots, and emerging trends of artificial intelligence are elucidated.

**Methods:**

On April 7th, 2022, articles and reviews regarding the application of AI in esophageal cancer, published between 2000 and 2022 were chosen from the Web of Science Core Collection. To conduct co-authorship, co-citation, and co-occurrence analysis of countries, institutions, authors, references, and keywords in this field, VOSviewer (version 1.6.18), CiteSpace (version 5.8.R3), Microsoft Excel 2019, R 4.2, an online bibliometric platform (http://bibliometric.com/) and an online browser plugin (https://www.altmetric.com/) were used.

**Results:**

A total of 918 papers were included, with 23,490 citations. 5,979 authors, 39,962 co-cited authors, and 42,992 co-cited papers were identified in the study. Most publications were from China (317). In terms of the H-index (45) and citations (9925), the United States topped the list. The journal “New England Journal of Medicine” of Medicine, General & Internal (IF = 91.25) published the most studies on this topic. The University of Amsterdam had the largest number of publications among all institutions. The past 22 years of research can be broadly divided into two periods. The 2000 to 2016 research period focused on the classification, identification and comparison of esophageal cancer. Recently (2017-2022), the application of artificial intelligence lies in endoscopy, diagnosis, and precision therapy, which have become the frontiers of this field. It is expected that closely esophageal cancer clinical measures based on big data analysis and related to precision will become the research hotspot in the future.

**Conclusions:**

An increasing number of scholars are devoted to artificial intelligence-related esophageal cancer research. The research field of artificial intelligence in esophageal cancer has entered a new stage. In the future, there is a need to continue to strengthen cooperation between countries and institutions. Improving the diagnostic accuracy of esophageal imaging, big data-based treatment and prognosis prediction through deep learning technology will be the continuing focus of research. The application of AI in esophageal cancer still has many challenges to overcome before it can be utilized.

## Introduction

Atypical hyperplasia and infiltration of the esophageal squid epithelium or glandular epithelium leads to the emergence of esophageal carcinoma (EC), which is mainly divided into two types, esophageal adenocarcinoma (EAC) and esophageal squamous cell carcinoma (ESCC), according to the proliferation of different epithelia. Globally, ESCC accounts for 84% of esophageal cancer cases, while EAC accounts for 15% (1, 2). EC is the seventh most common cancer (by incidence) and the sixth most lethal cancer (by mortality), with a fatality rate of more than 50% for new patients (3, 4). In terms of esophageal cancer incidence, industrialized and developing countries vary markedly (5–7). Esophageal cancers have a particularly dismal prognosis since they often generate no symptoms and are thus detected late in their progression. Resection and definitive cure are typically out of the question at this point. More than half of patients with esophageal cancer have distant metastases or unresectable illness (8). These factors result in a poor 5-year survival rate that, while rising with time, remains at only 18% (9).

Artificial intelligence (AI) has been established and has played a considerable role in the medical industry, owing to the emergence of deep-learning algorithms, computer hardware developments, and the exponential rise of data that is generated and used for clinical decision-making (10, 11). AI is defined as machine intelligence as opposed to genuine human intelligence. It is a branch of computer science concerned with creating a machine that can imitate human cognitive abilities such as learning and problem solving (12, 13). The two primary disciplines of AI in the medical profession are virtual and physical. Machine learning (ML) and deep learning (DL) are two fields of artificial intelligence (AI). Convolutional neural networks (CNNs), a type of deep neural network, are multilayer artificial neural networks (ANNs) that may be used to analyze images. Medical equipment and robotics are examples of physical AI (14). As more multidimensional data are created in normal esophageal cancer therapy and care, AI can assist doctors in developing a customized image of a patient throughout the progression of their care, eventually guiding therapeutic decisions. These decisions are based on the integration of different, complicated data streams, such as clinical presentation, patient history, esophageal carcinoma pathology, genetics, and endoscopic imaging, as well as the marriage of these data to the results of an ever-growing body of scientific literature. There is now a computational foundation for integrating and synthesizing these data to forecast where the patient’s treatment journey will lead and, eventually, enhance management decisions.

While there are many reasons to be optimistic, there are several obstacles to the successful integration of AI in clinical esophageal cancer. In terms of EC detection of premalignant and malignant lesions, while histopathologic examination is the gold standard for establishing the diagnosis of EC and determining the presence of dysplasia, endoscopists must collect targeted biopsies from particular areas that host the real lesion. AI can assist clinicians in performing directed biopsies rather than depending on random samples by detecting locations that may harbor Barrett’s esophagus with or without dysplasia; this AI assisted method of biopsy has been presented as a potential solution to the aforementioned problem (15, 16). Some esophageal cancer studies employed CNNs models as classifiers, while others used joint diagonalization principal component analysis (JDPCA), VGG16 Net, or Google Net. Although the values of accuracy, sensitivity, and specificity in esophageal SCC identification varied between studies, all models performed at least as well as endoscopists in lesion recognition and characterization, if not significantly better (17–21). Two studies by Nakagawa et al. (22) and Shimamoto et al. (23) used separate validation datasets to create models that predicted esophageal malignancy depth using a DL model based on a CNNs with a belief-propagation decoder. These models predicted invasion depth with an accuracy of 89.2% and 91%, respectively, with sensitivities of 70.8% and 90.1% and specificities of 94.4% and 95.8%. In terms of predictive ability, artificial neural networks (ANNs) were used by Mofidi et al. (24) to predict the survival rates of patients after surgical resection for the first year and third year with an accuracy of 88% and 91.5%, respectively as early as 2006. With the constant advancement of machine learning algorithms, Moghtadaei et al. (25) discovered in 2014 that early squamous dysplasia, a risk factor for esophageal cancer, is important for predicting the prognosis risk of postoperative patients and for clinical screening of high-risk groups using the least squares’ technique based on an evolutionary algorithm. Subsequently, a support vector machine (SVM)-based diagnostic model for esophageal cancer lymph node metastases was presented. To develop an SVM esophageal cancer lymph node metastasis prediction model, preoperative basic information and different index information on CT images of esophageal cancer patients undergoing radical chemotherapy were gathered. The area under the ROC curve was 0.887 (26). Chen et al. (27) developed a new diagnosis approach for esophageal squamous cell carcinoma (ESCC) in 2020, using a machine learning system with plasma metabolomics. The study combined plasma metabolomics with machine learning methods. For the early detection of ESCC, this new ESCC diagnostic approach can be applied.

Keeping up with the rapidly changing corpus of literature is vital not just because new discoveries occur from a wide range of fields, but also because new results may profoundly alter the collective knowledge of everyone researching AI (28). As interest in AI application research in the field of EC has grown rapidly, and a significant number of relevant papers have been published, it has become challenging for academics to identify the most recent advancements and research hotspots in this subject. According to current research, AI is still evolving quickly and is only in its early stages of application in the field of EC. The following research will benefit much from summarizing its worldwide research trends and research hotspots. However, no research on bibliometric analysis has been conducted to synthesize the literature in this domain. Bibliometric analysis (29–31), which has been widely used in many fields (32, 33), is an information visualization method for comprehending the knowledge structure and identifying the research frontiers or hotspots of a specific field by summarizing all of the literature in that field from around the world and quantitatively analyzing the literature data and metrological characteristics using mathematical and statistical methods. The data from the database may also be used to analyze and compare the research status of other nations, institutions, and authors, so that we can better comprehend scientific publications and better illustrate the research patterns (34–36).

Here, we determine the countries, institutions, authors, or journals with the highest citations/publications of AI in the field of EC by collecting literature data in the database, and therein describe the challenges faced in the EC clinical translation of AI. The aim of this study is to characterize the application and progress of AI in EC from 2000 to 2022 utilizing bibliometric analysis and to identify the current research progress, hotspots, and emerging trends of AI in EC, which may assist new researchers comprehending future research and identifying areas of interest for further research.

## Methods

### Database

The data source was The Science Citation Index Expanded (SCI-Expanded) of Clarivate Analytics’ Web of Science Core Collection (WoSCC). Web of Science applies a strict screening process. Bradford’s Law in bibliometrics states that only prominent academic publications from many areas are included. SCIE, as a journal citation subdatabase of WoSCC, is a multidisciplinary comprehensive database covering the field of natural science, with over 8,600 global authoritative journals encompassing 176 topic categories.

### Search strategy

To guarantee that no data updates were made, two researchers from our organization examined the information of papers concerning AI in the field of EC simultaneously and finished the search in one day. The articles’ titles, keywords, abstracts, authors, institutions, and reference data were obtained and stored in plain text format. The following was the search formula:

1#: esophag* (Topic) or oesophag* (Topic) or gullet (Topic) and Article OR Review (Document Type) and English (Language) [103,423results]

2#: cancer* (Topic) or tumor* (Topic) or tumor* (Topic) or neoplas* (Topic) or onco* (Topic) or carcinoma* (Topic) and Article OR Review (Document Type) and English (Language) [3,272,272 results].

3#: 1# AND 2# [54,077 results]

4#: artificial intelligent* (Topic) or computational NEAR/5 intelligence (Topic) or expert* system* (Topic) or intelligent learning (Topic) or feature* extraction (Topic) or feature* mining (Topic) or feature* learning (Topic) or machine learning (Topic) or feature* selection (Topic) or unsupervised clustering (Topic) or image* segmentation (Topic) or supervised learning (Topic) or semantic segmentation (Topic) or deep network* (Topic) or bayes* network (Topic) or deep learning (Topic) or neural network* (Topic) or neural learning (Topic) or neural nets model (Topic) or artificial neural network (Topic) or data mining (Topic) or graph mining (Topic) or data clustering (Topic) or big data (Topic) or knowledge graph (Topic) or AI (Topic) and Article OR Review (Document Type) and English (Language) [1,068,667 results].

5#: 3# AND 4# [1,074 results].

### Data analysis and visualization

Two researchers independently analyzed the data to ensure the accuracy of the data and the repeatability of the research. Microsoft Excel 2019 was used to analyze and export the files of top-cited or productive authors, countries/regions, publications, journals, and institutions. H-index was a hybrid index proposed by Hirsch that can be used to evaluate academic achievements (37). Altmetrics, which was introduced in 2012, is a supplemental statistic used to monitor reader behaviors as well as interactions with content and social media (38, 39).

CiteSpace is a popular information visualization method in the field of knowledge graphs (40). This review uses CiteSpace 5.8.R3 (64-bit) to accomplish visualization to obtain insights into the application of AI on EC and identify the research horizon and knowledge base of the field in large amounts of data. The most often employed metrics in bibliometric analysis are co-authorship, co-citation, and co-occurrence analysis (41–43). The purpose of co-authorship analysis is to examine the link between authors, nations, or organizations based on the number of articles produced together. Co-occurrence analysis is a quantitative tool for analyzing the connection between several objects based on whether they appear together. Co-citation analysis demonstrates the degree of the association between cited things based on the number of citing items (44–46). Significantly, when the clustering function was activated, the Modularity Q and Mean Silhouette scores had a significant influence on visualization, indicating an overall structural feature of the network. Overall, Q > 0.3 revealed a strong structure. If S was more than 0.5, the cluster considered logical (47).

The VOSviewer is a scientific knowledge graph application that can depict the structure, progression, coordination, and other aspects of knowledge fields by constructing linkages and visually analyzing literary knowledge items (48). In this research, citation/co-citation and keyword cooccurrence analyses were performed. In addition, for country/region co-authorship and publication analyses, an online analytic platform (http://bibliometric.com/) and the bibliometrix R package for bibliometric analysis were used. The calculation of the almetrics is performed as “Almetric Attention Scores” through a free browser plugin provided by Almetric (https://www.altmetric.com/).

### Research ethics

The data used in this study were acquired from an open source and do not require approval by any ethical committee.

## Results

### Basic statistical analysis

#### Global trends of publication and citation

**Figure 1:**
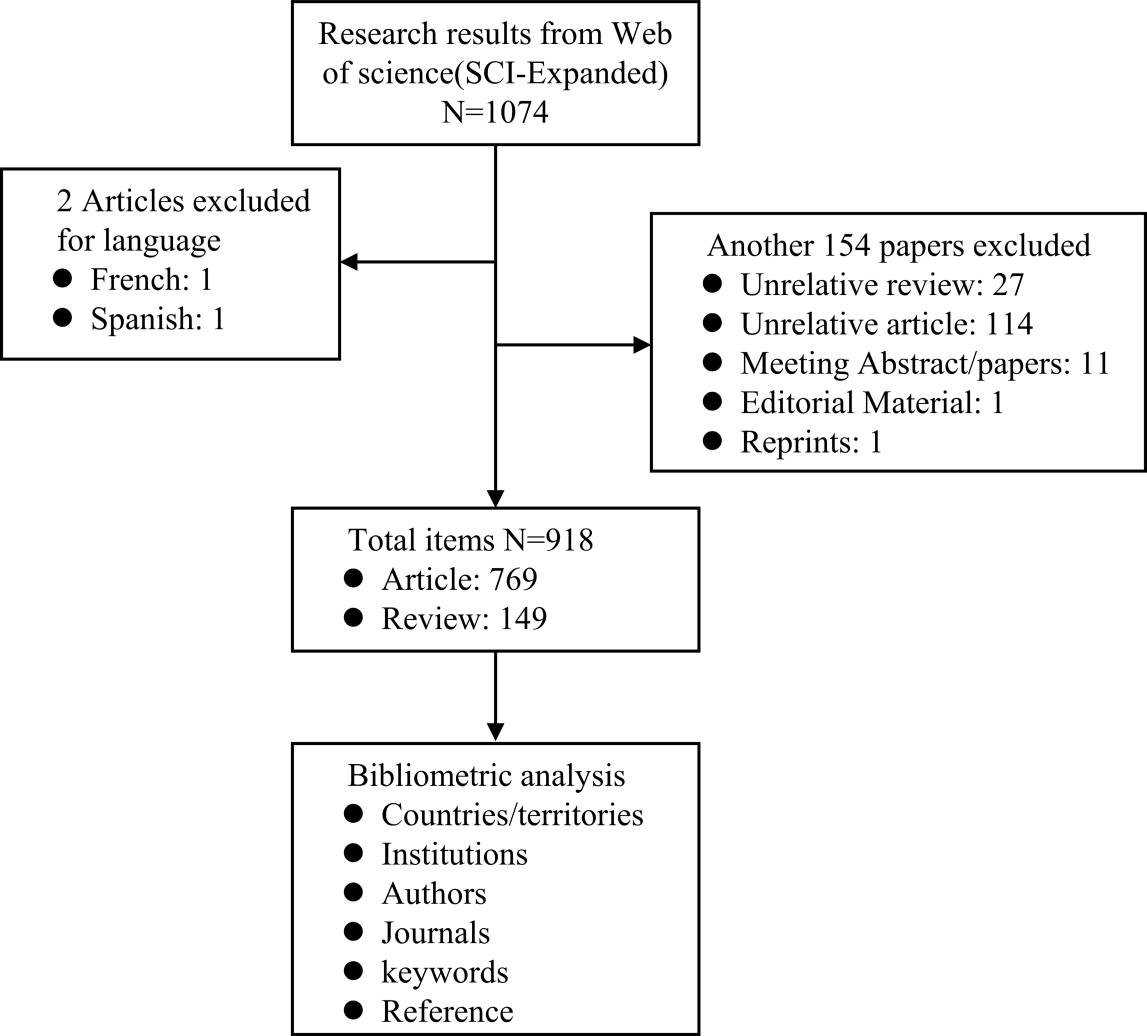
Flowchart of the study strategy.

**Figure 2:**
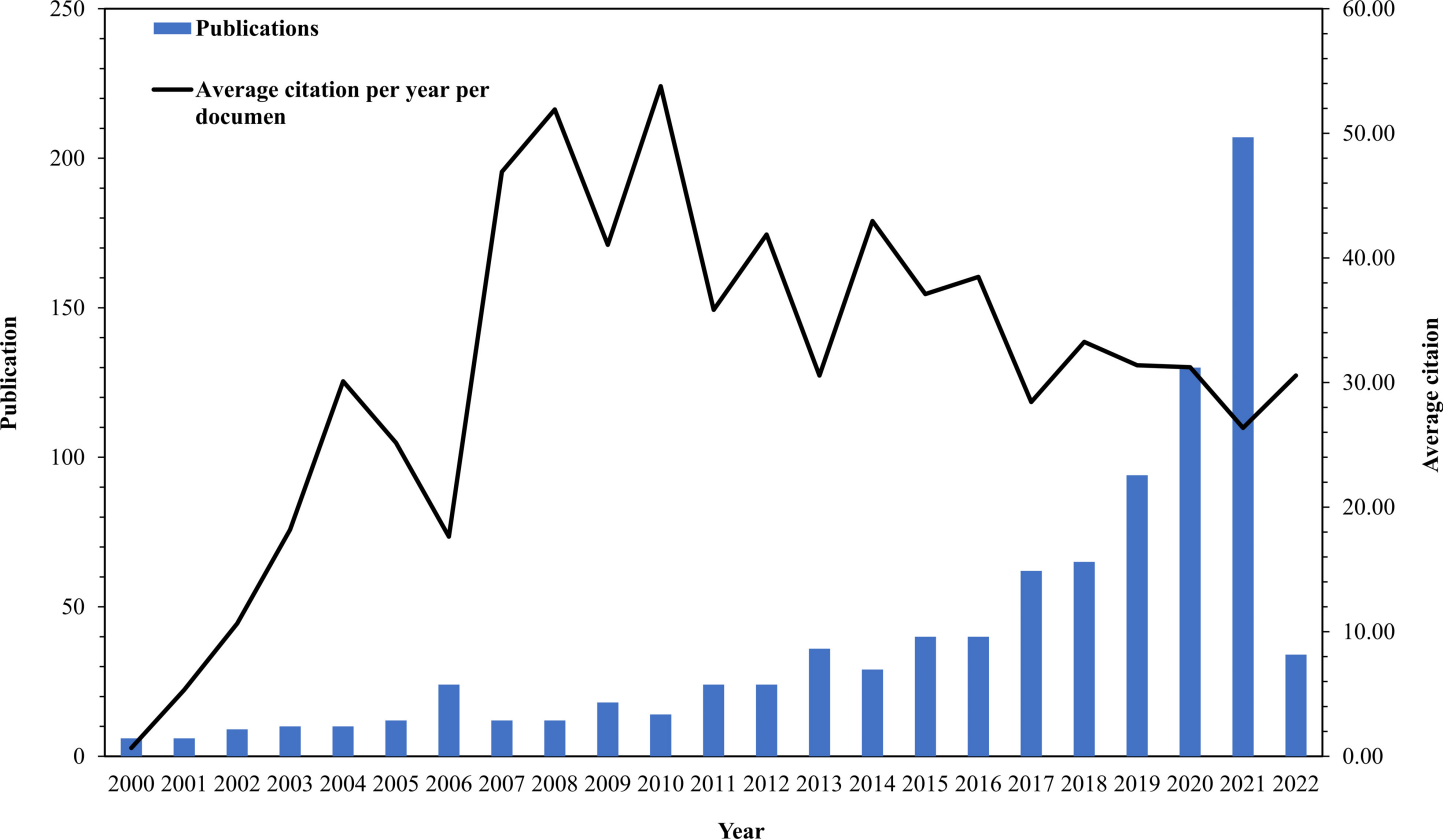
Global trend of publications and average citation on artificial intelligence research in esophageal carcinoma (2020-2022).

As per the study strategy flowchart, we eventually gathered 918 publications from the Web of Science (SCI-E) panning the previous 22 years, comprising 769 articles and 149 reviews (**Figure 1**). **Figure 2** shows that since 2000, AI research in EC has continuously expanded year after year. The number of published papers was small during the earlier years that were analyzed. Meanwhile, the average number of citations each year per document was not stable, and the range of variation was spacious. Over the last decade, research has advanced quickly, accounting for more than 80% of all publications. The growth rate over the last 6 years has resembled exponential growth, whereas average citations have remained approximately 30. The phenomena demonstrate that the application of artificial intelligence in esophageal cancer research is gaining traction, and the quality of papers in the field has been improved. As of the search date (April 10, 2022), all papers have been cited 23,490 times, with an H-index of 74 and an average citation count of 25.37.

#### Contributions of top productive countries/regions

**Figure 3:**
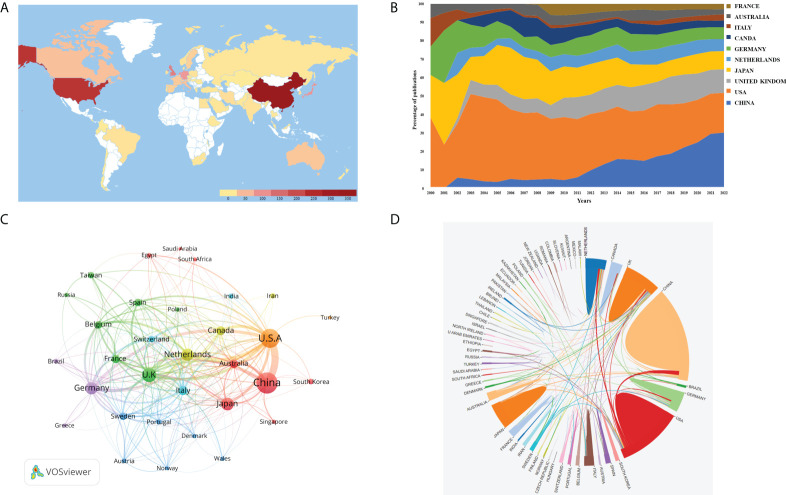
**(A)** World map based on the total publications of different countries/regions. **(B)** The changing consistence of the annual publication quantity in the top 10 countries/regions from 2000 to 2022. **(C)** The countries/regions' citation networkvisualization map generated by using VOSviewer. The thickness of the lines reflected the citation strength. **(D)** The international collaborations' visualization map of countries/regions. The thickness of the line between countries reflects the frequency of the cooperation.

In this category, 53 countries/regions have published relevant publications. According to the global map in **Figure 3A**, nations that have produced more than 200 papers included the United States and China. **Figure 3B** depicts the publishing tendencies of the top ten nations over the last 22 years. **Supplementary Table 1** shows that China is placed at the top (with 317 articles). However, the United States ranked top in overall citations (9,927 times), outnumbering China, which ranks second (4,153 times). We applied VOSviewer to examine the collaboration (**Figure 3C**). When the minimum number of articles was set at higher than 5, 31 nations were included. The lines connecting nodes represent co-authorship between countries, and the thicker the line is, the stronger the collaboration. This co-authorship visualization map revealed that the top five TLS (total link strength) countries were the United States, the United Kingdom, the Netherlands, Germany, and China. As seen in **Figure 3D**, the United States had the closest collaboration with numerous nations, the most significant of which were China, the Netherlands, and the United Kingdom. Other countries’ collaboration, on the other hand, was fragile.

#### Contributions of top journals

**Figure 4:**
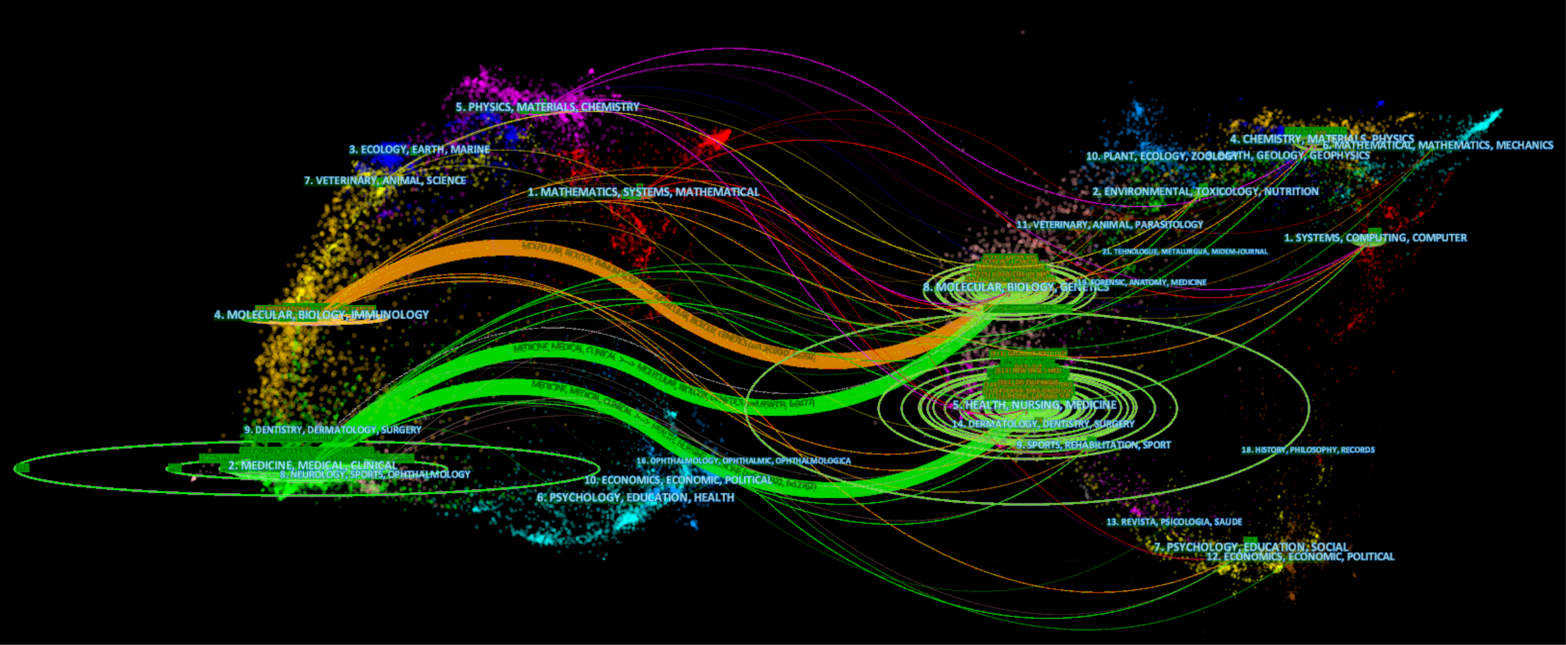
A dual-map overlap of journals on AI research in EC carried out by Citespace.

All of the papers were published in a total of 638 journals, with 378 of the journals publishing at least 10 articles. **Table 1** shows that the top three most productive journals were the New England Journal of Medicine (324, 30.29%), Gastroenterology (300, 32.68%), and Gut (269, 29.30%). Furthermore, the total number of citations in the New England Journal of Medicine was 464,376, which was much higher than in other publications. **Table 1** represents the best ten journals that published the most articles on AI on EC between 2010 and 2022. The New England Journal of Medicine ranked highest with approximately 1,030 publications. In general, the topic scope includes Gastroenterology, Hepatology, Oncology, Medicine, General & Internal Medicine, Multidisciplinary Sciences, and so on. Ca-A Cancer Journal for Clinicians has the greatest impact factor among the top ten journals, with approximately 508.7. Eight of the top ten journals listed in **Table 1** were located in Q1. **Figure 4** shows the link between citing and cited journals using a dual map of journals. It was clear that there were primarily three citation paths (1): Molecular, Biology and Immunology—Molecular, Biology, Genetics; (2) Medicine, Medical, Clinical—Molecular, Biology and Immunology; (3) Medicine, Medical, Clinical—Health, Nursing, Medicine. The citing papers are mainly concentrated in 3 circles including 3 fields (1) Molecular, Biology and Immunology; (2) Neurology, Sports, Ophthalmology; and (3) Medicine, Medical, Clinical. The cited papers were mainly located in 4 circles containing 6 fields (1) Health, Nursing, Medicine; (2) Dermatology, Dentistry, Surgery; (3) Molecular, Biology, Genetics; (4) Chemistry, Materia, Physics (5) Mathematical, Mathematics, Mechanics; and (6) Systems, Computing, Computer.

**Table 1:** Top 10 journal published analysis concerning the research of AI on EC (2000 - 2022).

Rank	Journal Title	Country	Count	IF (2020)	JCR (2020)	Research Area	H-index
1	New England Journal of Medicine	USA	324	91.25	Q1	Medicine, General & Internal	1,030
2	Gastroenterology	UK	300	22.68	Q1	Gastroenterology & Hepatology	402
3	Gut	UK	269	23.05	Q1	Gastroenterology & Hepatology	293
4	Gastrointestinal Endoscopy	USA	244	9.43	Q1	Gastroenterology & Hepatology	200
5	PloS One	USA	240	3.24	Q2	Multidisciplinary Sciences	332
6	Ca-A Cancer Journal for Clinicians	USA	235	508.7	Q1	Oncology	168
7	Journal Of Clinical Oncology	USA	234	24.01	Q1	Oncology	548
8	International Journal of Cancer	Switzerland	222	7.396	Q1	Oncology	234
9	Technology In Cancer Research & Treatment	USA	215	3.34	Q4	Oncology	63
10	Nature	UK	211	49.962	Q1	Multidisciplinary Sciences	1,226

#### Analysis of institution and co-institution

**Figure 5:**
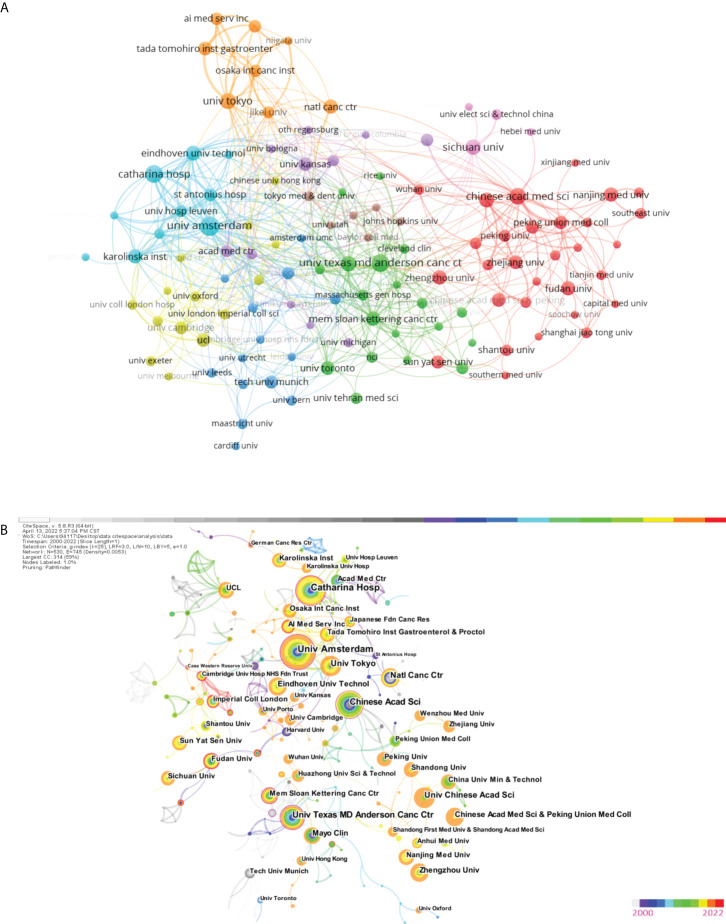
**(A)** The citation network visualization map of institutions was performed with VOSviewer. **(B)** Co-institutions' network (2000-2022). The color of the circle represents when the article was published. The larger the node diameter, the more papers institutions have published. The thicker the line between the nodes, the closer the two institutions work together. The outermost purple circle indicates that this institution has a very strong intermediary role in the field (centrality>0.1).

More than 118 universities played an active role in AI application research at the European Commission, with the top three TLSs being the University of Amsterdam (TLS = 72), Catharina Hospital (TLS = 64), and Eindhoven University of Technology (TLS = 53). **Table 2** outlines the top ten institutions with the largest contribution, with the leading three being the University of Amsterdam, Memorial Sloan Kettering Cancer Center, Catharina Hospital, and Chinese Academy of Sciences, with 25, 22, and 20 papers, respectively. Nevertheless, most institutions were dispersed and there was insufficient partnership, with most partnerships having performed at American and Chinese universities (**Figure 5A**). We launched Cite Space and generated a network as usual: 2000-2022, 1 year slice length Node Choose a node type: Institution, g-index (k = 25), Pathfinder selection, slice time pruning, and combined network pruning. Other parameters were set to their default values.

**Table 2:** Top 10 institutes in the publications concerning the research of AI on EC.

Rank	Institutions	Countries/regions	Counts	TLS	Total citations
1	University of Amsterdam	Netherlands	25	72	879
2	Catharina Hospital	Netherlands	22	64	581
3	Chinese Academy of Sciences	China	20	29	714
4	The University of Texas MD Anderson Cancer Center	USA	18	39	536
5	University of Chinese Academy of Sciences	China	15	14	373
6	University of Tokyo	Japan	15	53	756
7	National Cancer Centre Singapore	Singapore	14	9	450
8	Zhengzhou University	Zhengzhou	13	12	544
9	Chinese Academy Medical Science & Peking Union Medical College	Beijing	13	7	22
10	Eindhoven University of Technology	Netherlands	13	53	238

In addition, the Co-institutions knowledge mapping was constructed, with N = 530 and E = 745. (Density was 0.053). **Figure 5B** reveals which universities have the most research strengths. The outermost purple circle indicates that Chinese Academic Science, University of Amsterdam, Memorial Sloan Kettering Cancer Center, Saint Antonius Hospital, Catharina Hospital, Cambridge University Hospitals NHS Foundation Trust, University of Texas MD Anderson Cancer Center, Univ Maryland, Duke University, and University of North Carolina play a major role in the study of AI in EC. Their centrality values are 0.19, 0.18, 0.17, 0.16, 0.14, 0.14, 0.12, 0.11, 0.10, and 0.10.

#### Analysis of author and co-author

**Figure 6:**
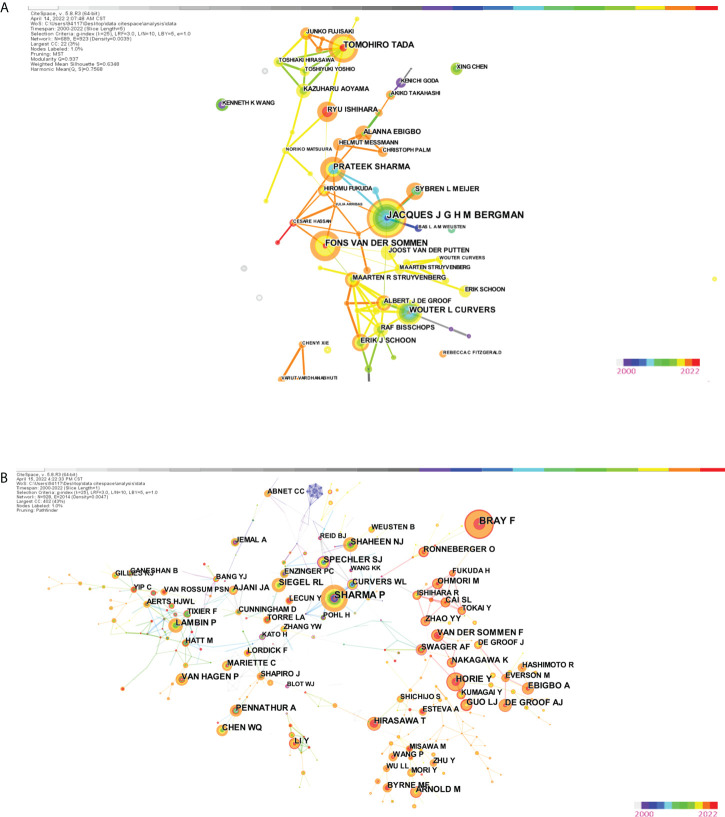
**(A)** The visualization map of co-authorship carried on CiteSpace. **(B)** The visualization map of co-citation (cited author) carried on CiteSpace.

The research includes 5 979 authors and 39,962 co-cited writers. **Table 3** displays the top ten most prolific writers as well as the top ten co-cited authors with the most citations. Tewari Ashutosh K, Menon Mani, and Patel Vipul R were in the top three, with 66, 54, and 51 articles, respectively. **Figure 6A** demonstrates that the author’s centrality was less than 0.1 and that only a few interconnections were visible in the author’s cooperation network map. The betweenness centrality (BC) of a node is an indication of its centrality that can show the importance of nodes in networks. In general, nodes with BC values greater than 0.1 hold important places linking a significant number of nodes and are typically characterized as hub nodes, which are depicted in purple rings (49).

**Table 3:** The 10 most productive authors and the top 10 co-cited authors with the highest citations.

Rank	Author	Country	Count	Total citations	Co-cited author	Country	Count	Total citations	Centrality
1	Jacques J G H M Bergman	Netherlands	16	459	Freddie Ian Bray	France	89	304	0.00
2	Tomohiro Tada	Japan	12	384	Prateek Sharma	USA	87	1907	0.04
3	Fons Van Der Sommen	Netherlands	12	203	Yoshimasa Horie	Japan	56	691	0.04
4	Wouter L Curvers	Netherlands	10	403	Jacques Ferlay	France	53	748	0.01
5	Prateek Sharma	United States	10	303	Jesper Lagergren	Sweden	52	1591	0.15
6	Ryu Ishihara	Japan	8	94	Lambin Philippe	Belgium	46	562	0.04
7	Sybren L Meijer	Netherlands	7	140	Rebecca L Siegel	USA	46	417	0.00
8	Erik J Schoon	Netherlands	7	195	Hirasawa Toshiaki	Japan	45	1025	0.06
9	Alanna Ebigbo	Germany	7	53	Nicholas J Shaheen	USA	45	136	0.04
10	Raf Bisschops	Belgium	6	189	Thomas William Rice	USA	43	1107	0.12

Mani Menon, Ficarra V, and Tewari Ashutosh K had the most citations in a co-cited author network study. Tewari A’s and Kattan MW’s BC were as high as 0.4 and 0.35, respectively, showing that their contributions had a significant impact in this sector. The modularity Q value was used to assess the network’s clustering impact. The higher the value is, the better the network’s clustering performance. The silhouette value, which was used to quantify network homogeneity, was another indication. The modular Q value was 0.7218, and the mean silhouette S value was as high as 0.9248, as shown in **Figure 6B**, showing that the clustering effect and network homogeneity were reliable.

### Analysis of cited references

**Figure 7:**
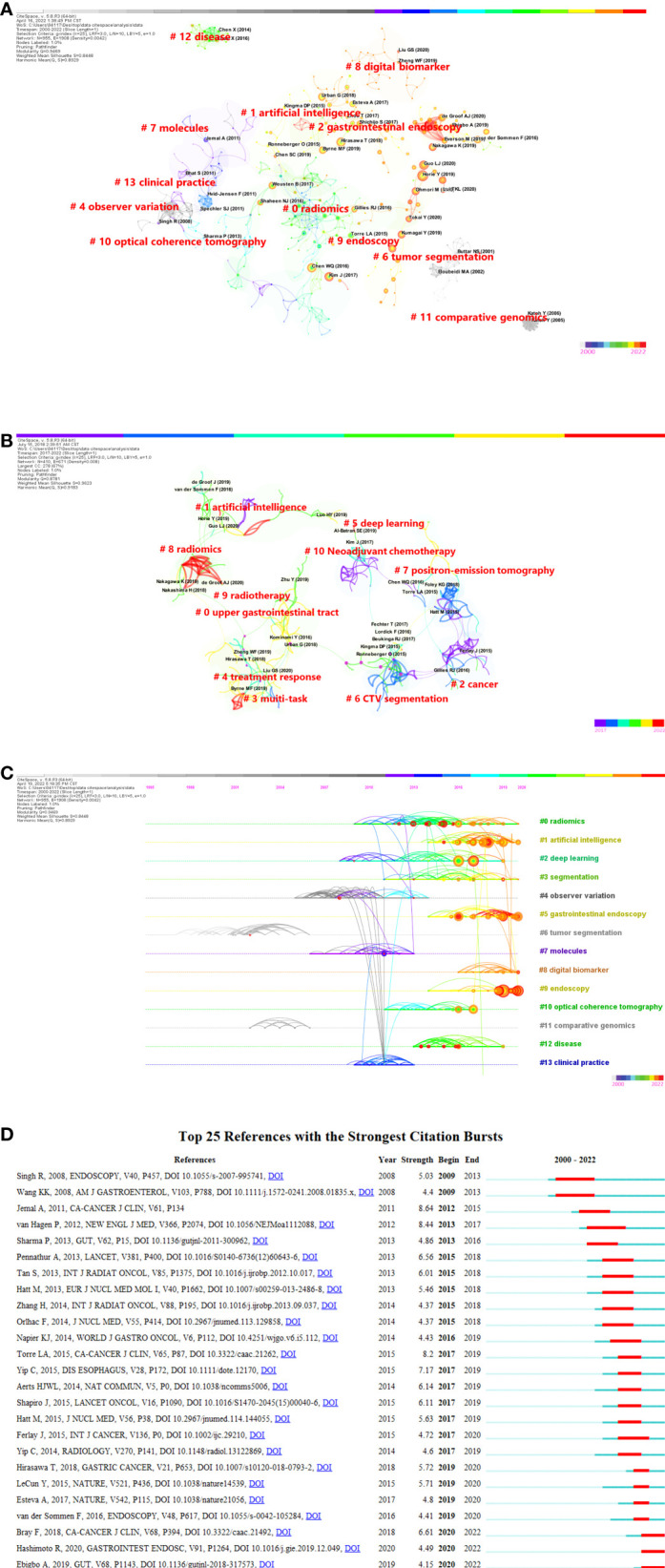
**(A)** Citespace visualization map of cluster view (cited references) **(B)** A landscape view of co-cited reference cluster analysis from 2017 to 2022. **(C)** CiteSpace visualization map of timeline view. The time evolution is indicated with different colored lines, and the nodes on the lines indicate the references cited. **(D)** CiteSpace visualization map of top 25 references with the strongest citation bursts from 2000 to 2022.

This analysis included a total of 918 publications, 80 of which were cited at least ten times. Horie Y et al. (50) had the highest total citation frequency, as stated in **Table 4**, with 61 citations (Local). Guo LJ et al. (51) came in second place with 41 citations. Since an article’s almetrics are mostly determined by its network exposure. Greater network influence is indicated by higher scores. With Almetric Attention scores of 57, the article by the author of De Groof AJ et al. (52) is the most influential in social media and other networks, having been referenced by 6 news sites, 17 tweeters, and 2 Facebook pages. The co-citation network analysis of references is depicted graphically in **Figure 7A**. According to the analysis results, the Modularity Q was 0.9469, and the mean Silhouette S was 0.8448, indicating an outstanding clustering effect and strong network homogeneity. Given that the majority of the included papers were published within the previous 6 years, we used co-cited reference clustering based on the most recent publications to better identify the research fronts in **Figure 7B**. The Modularity Q and mean Silhouette S both demonstrated a great clustering effect and network homogeneity. Finally, we obtained 11 clusters that clearly demonstrate the hotspots and cutting-edge content of artificial intelligence in the area of esophageal cancer in recent years. More than half of the clusters “treatment response” (#4), “CTV segmentation” (#6), “positron-emission tomography” (#7), “radiomics” (#8), “radiotherapy” (#9), and “neoadjuvant chemotherapy” (#10) are relevant for the accurate diagnosis and treatment of esophageal cancer. **Figure 7C** also depicts a timeline view of the co-citation references, which reflects the evolution of research hotspots through time. The clustering findings revealed that it could be classified into 38 groups, yet only the top 14 were shown in **Figure 7A**. The largest cluster was “radiomics” (#0) (53–56), while “tumor segmentation”(#6) (57–61)was the earliest research in this field. “Endoscopy” (#9) (62–65) and “deep learning” (#2) (66–70) were the latest research hotspots. **Figure 7D** exhibits the top 25 references with the strongest citation bursts. The citation eruption in this discipline began in 2009.

**Table 4:** Top 10 local cited documents concerning the research of AI on EC.

Rank	Author		Journals	DOI	Year	Local Citations	Almetric Attention Scores
1	Horie Y; et al.	2019	Gastrointestinal Endoscopy	10.1016/j.gie.2018.07.037	2019	61	12
2	Guo LJ; et al.	2020	Gastrointestinal Endoscopy	10.1016/j.gie.2019.08.018	2020	41	12
3	De Groof AJ; et al.	2020	Gastrointestinal Endoscopy	10.1053/j.gastro.2019.11.030	2020	36	57
4	Ohmori M; et al.	2020	Gastrointestinal Endoscopy	10.1016/j.gie.2019.09.034	2020	35	5
5	Van Der Sommen F; et al.	2016	Endoscopy	10.1055/s-0042-105284	2016	34	51
6	Cai SL; et al.	2019	Gastrointestinal Endoscopy	10.1016/j.gie.2019.06.044	2019	34	16
7	Zhao YY; et al.	2019	Endoscopy	10.1055/a-0756-8754	2019	32	1
8	Nkagawa K; et al.	2019	Gastrointestinal Endoscopy	10.1016/j.gie.2019.04.245	2019	31	17
9	Hashimoto R; et al.	2020	Gastrointestinal Endoscopy	10.1016/j.gie.2019.12.049	2020	30	29
10	Tokai Y; et al.	2020	Esophagus-Tokyo	10.1007/s10388-020-00716-x	2020	29	0

### Analysis of keywords

**Figure 8:**
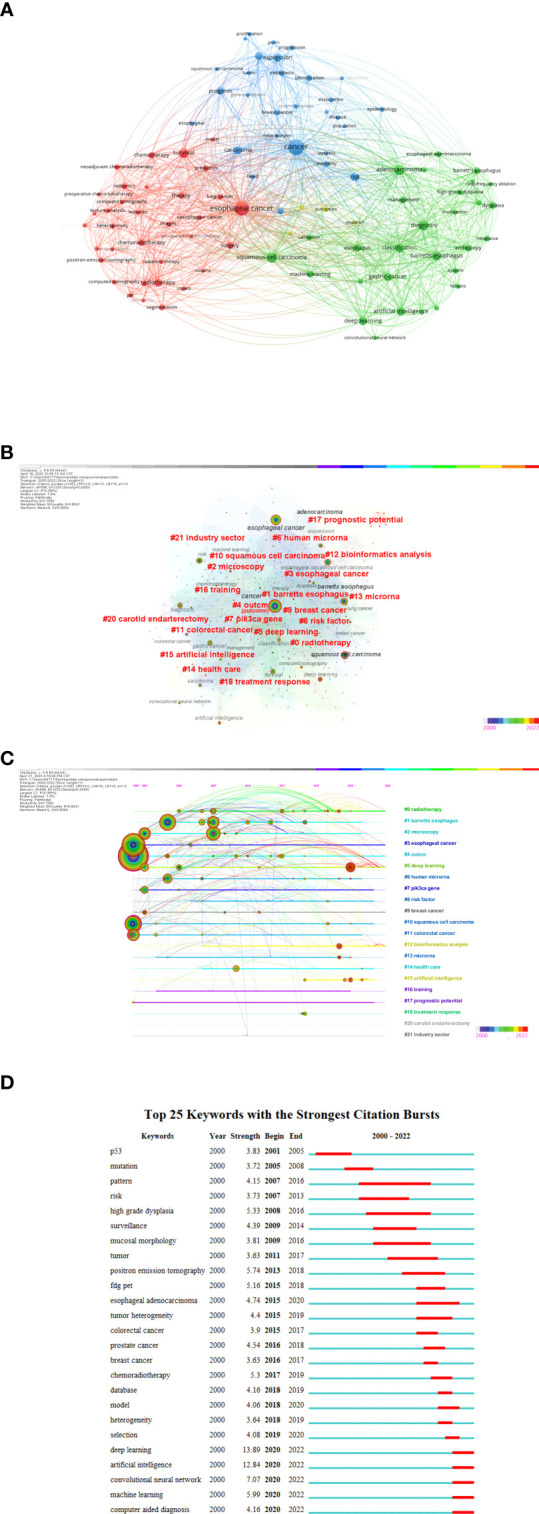
**(A)** The network visualization map of the 96 keywords with a frequency of no less than 15 times generated by using VOSviewer. **(A)** All the keywords could be clustered into 3 main clusters: #Cluster 1 (Cancer-AI-related study, red nodes), #Cluster 2 (Esophageal cancer AI-related study, blue nodes), and #Cluster 3 (Adenocarcinoma AI-related study, green nodes). **(B)** A landscape view of keyword cluster analysis generated by g-index (K = 25) per slice from 2000 to 20222. (LRF = 3.0, L/N = 10, LBY = 5, and e = 1.0). **(C)** CiteSpace visualization map of timeline view. The time evolution is indicated with different colored lines, and the nodes on the lines indicate the keyword clusters appearance. **(D)** CiteSpace visualization map of keywords with the strongest citation bursts from 2000 to 2022.

Keyword frequency analysis clarifies the research patterns in this study. As seen in **Figure 8A** by the VOSviewer, cancer, esophageal cancer, and adenocarcinoma had frequencies of more than 100 times, and squamous-cell carcinoma, diagnostic, survival, Barrettes-esophagus, classification, deep learning, risk, artificial intelligence, expression, and other related terms were reasonably high with frequencies of over 50 times. We practiced CiteSpace to create a network. The nodes were revised based on the co-occurrence of keywords, and the log-likelihood (LLR) algorithm was used to calculate clustering. That can be seen in **Figure 8B**, the Modularity Q score was 0.7682 and the Mean Silhouette score was 0.8941. There were a total of 22 clusters, as listed in **Supplementary Table 2**.

Clusters are characterized along a horizontal timeline in a timeline visualization. **Figure 8C** illustrates the 22 clusters. Each one may show the progress of AI research on EC from 2000 to 2022. The most recent hotspots in this area were “deep learning” (#5) (71). Citation bursts are terms that occur abruptly in a short period of time or whose usage frequency dramatically increases.

Generally, citation bursts indicates the evolution of the study issue over time, just as shown in **Figure 8D**. The term eruption in this field began in 2001, showing that the use of artificial intelligence in the field of esophageal cancer has been drawing interest for more than 20 years. It can be seen from the figure that the salient intensity of deep learning is the highest, suggesting that future research on artificial intelligence in esophageal cancer will be carried out with deep learning.

## Discussion

Artificial intelligence (AI) research has accelerated in the previous 22 years, with clinical applications being examined for the majority of medical professions. The discipline of EC, which is highly dependent on imaging studies, is no exception. Bibliometric analysis, as opposed to systematic review, uses visual tools to completely examine the current literature to intuitively comprehend the development pattern of research and identify future research hotspots. This is the first studies to use bibliometric analysis to summarize the contemporary use of AI in EC and intuitively illustrate the development trend and future research hotspots by applying two commonly used literature measurement software tools: VOSviewer and CiteSpace.

The trajectory of the average citations of each article every year over the last 22 years allows us to see changes in the volume and quality of AI research in the field of esophageal cancer. The instability of the average citations per paper per year in the early days has developed into a relatively stable fluctuation range in the past 6 years, which suggests that the development of the entire discipline is maturing. China contributed the most to total publishing volume of any country (**Supplementary Table 1**). The number of contributions worldwide is growing year by year, indicating that China places a significant importance on scientific research in this domain (**Figure 3B**). Although China ranks first in terms of the number of publications, the H-index of China was only 29, with total citations of 4,425, even lower than that of France (H-index=12, 1,665 cited), indicating that, while the amount of literature in China has increased, it still lacks high-quality articles, and the main reason for this may be that AI research in China started late, with an average publication of 2018.68. Publication volume is followed by the United States, it has the highest H-index, which shows that United States publication has a greater impact around the world. According to the nation contact map based on WOSCC data, the United States has relations with numerous countries that are engaged in this domain, including China and the Netherlands (**Figure 3D**).

The top 3 publishing journals (**Table 1**) were the New England Journal of Medicine (IF = 91.25, Q1), Gastroenterology (IF = 22.68, Q1), and Gut (IF = 23.05, Q1). The impact factor (IF), JCR category, and total citations are useful indices of journal quality. Furthermore, the overall citations of the New England Journal of Medicine greatly outnumber those of other publications, confirming the journal’s significant importance in this field. More studies on the application of AI in EC are expected to be preferentially published in the aforementioned journals in the future. Furthermore, Gastroenterology, Gut, and Gastrointestinal Endoscopy were high-yield journals with the potential to produce additional high-quality papers in the future to increase their academic reputation and impact factor. The citing papers are mostly concentrated in three circles with three fields, whereas the cited papers are mostly concentrated in four circles with six fields. This finding implies that advancement in the field will require cross-disciplinary collaboration. Furthermore, scholarly interest has steadily increased.

The findings of research collaborations were also considerably impacted by nation. The Netherlands had 3 of the top 10 institutions (**Table 2**). This finding conveys that the Netherlands’ research in this subject is becoming increasingly relevant, and that it has emerged as a key research center. TLS is a measure of the closeness of collaboration. The top 3 strongest TLSs are held by the University of Tokyo and Eindhoven University of Technology. With the exception of China, the top ten most productive nations were developed countries, indicating that research on the use of AI in EC in developing countries was clearly falling behind that in developed countries. As a result, we believe that China should aggressively maintain strong cooperative contacts with other nations, and benefit from the superior technology and research techniques of other developed countries, in order to increase its impact in this field. Additionally, the majority of institutions were distributed with a density of 0.0001 (**Figure 5B**), indicating a lack of international coordination among institutions.

Furthermore, co-authorship analysis revealed that the BC value of each author was essentially less than 0.1, indicating that even though a large number of scholars participated in the study, they were relatively separated. In terms of co-cited authors, Jesper Lagergren has a BC value of 0.14, indicating the relevance of nodes within research networks. He was mainly engaged in the causes, prevention and treatment of esophageal and gastric cancer and related disorders, inputting data from multicenter for modelling to predict cancer-specific mortality and published a vast number of publications, demonstrating his significant influence in this subject (72). Thomas William Rice was another with a high BC value of 0.12 who was mostly involved in his primary research interests in clinical thoracic surgery (esophagus). It was discovered that staging esophageal and esophagogastric junction tumors for clinical use is quite significant (73).

The top 10 most cited publications reflect research hotspots and priorities in the field of artificial intelligence applied in EC. The majority of papers are concerned with the diagnosis of premalignant or malignant lesions (esophageal cancer in Barrett’s esophagus), the creation of objective scoring systems for risk stratification, forecasting disease prognosis, or therapeutic response. Co-citation analysis is frequently used to assess an author’s academic influence. As shown in **Table 4**, the most cited Chinese article in this study was Guo LJ et al. (51), who primarily introduced a specially developed system for computer-assisted diagnosis (CAD) for real-time automated diagnosis of precancerous lesions and early esophageal squamous cell carcinomas (ESCCs) to assist in the diagnosis of esophageal cancer. At the same time, we cannot disregard a paper’s review by networks such as social media. It is also commonly known that news spreads quickly on social media. The Almetric Attention Scores can be used to analyse this impact. De Groof AJ et al. (52) is the most influential in the network of these ten papers. This article, with the appealing title “With Higher Accuracy Than Endoscopists……” may be the explanation for its high Almetric Attention scores. It should be highlighted that public interaction or trends might be contentious in regard to establishing scientific merit or making policy changes.

The changes in research fronts can be seen through the timeline view of clustering results of co-cited references (**Figures 7A, C**). The earliest research laid emphasized on “tumor segmentation”, diagnosis, and then turned to the use of “deep learning”, “endoscope” “gastrointestinal endoscope” and other technologies. This change indicates that the early-stages of research in this field mainly focused on the classification and segmentation of esophageal tumor categories, then turned to the use of new techniques to improve the accuracy and efficiency of diagnosis and achieve early diagnosis. According to the citation analysis in this field (**Figure 7C**), it first burst in 2009, which suggests that the application of AI in esophageal cancer has just begun in the last decade and a large number of co-citation references are still being frequently cited. This indicates that AI research on esophageal cancer will be a hot spot in the future, especially in the area of esophageal endoscopic research (74). We may learn about the specific study contents during the last 6 years from the results of extensive analysis (**Figure 7B**). It mostly consists of two directions. On the one hand, endoscopic imaging or special endoscopy was utilized in these studies to determine dysplasia. The most common analytical models currently are neural networks and support vector machines. Cross-validation has been widely used as a validation strategy in these studies. Deep learning, as a rising star, obtained a discriminating accuracy of approximately 90% in the research of De Groof AJ et al. (52). They trialled a deep-learning computer-aided detection (CAD) system for boosting endoscopic detection of early neoplasia in patients having Barrett’s esophagus (BE). In general, such models outperformed nonprofessional endoscopists in distinguishing between normal and dysplastic/tumor images. On the other hand, as diagnostic technology advances, the discussion over precision therapy has improved. The creation of the first treatment approach has a direct impact on the management of esophageal cancer patients. A typical dilemma is determining which combination of systemic medicine, radiation therapy, and surgery is optimal for patients with various stages of esophageal cancer. Predicting radiation sensitivity will help in the development of this method. Suitably, deep learning has been demonstrated to be capable of analysing multidimensional data streams in the genomics area and making effective radiation sensitivity predictions on data incorporating radiometric indications. This finding marks the main direction of the current research on the application of artificial intelligence in esophageal cancer.

The study of the frequency of keywords may reflect the development tendency of research hotspots from another point of view, which further confirms the findings of this study. As shown in **Figure 8A**, we classified all keywords into three clusters, named “cancer”, “adenocarcinoma” and “esophageal cancer”. Based on the number of articles published in each year (**Figure 2**), we divided the keywords into two periods for analysis (1). 2000-2016 The early stage of field development was a period of delay. At this time, research was mainly concerned with about the analysis of total cancer types (a part of it, gastrointestinal tumor), which paid more attention to biological markers (75), outcome (76), and risk factor (77) among others. However, the analytical techniques used were limited, and data analysis was still at a small-scale and superficial level. This scope of research was different from the AI research in the field of other cancers such as prostate cancer, which mainly focused on cancer screening methods and surgical treatment methods (78) (2). 2017-2022 These six years represent a period of explosive growth in the number of published articles. Computer-aided diagnosis (79) and computer-aided therapy (80) have become the main application directions, and deep learning (#5) has emerged as the name of specific methods of artificial intelligence with the highest word frequency13.89 (**Figure 8D**). Specifically, deep learning plays a role in early detection (81), accurate differentiation of precancerous lesions from tumor lesions (82), determination of invasive tumor margins during surgical treatment (83), monitoring of disease progression and acquired drug resistance (84), and prediction of tumor aggressiveness (85), metastasis pattern (86) and recurrence risk (87). The innovation of esophageal imaging recognition and cancer marker screening technology provides the possibility for esophageal cancer detection, treatment and monitoring. Deeper technical levels of AI at this stage come into play. The application of AI in the field of esophageal cancer shows an overall delay. After a delay of at least 10 years, the exploration of the application of AI in EC has been carried out in the same way as in other cancers. The reason for this phenomenon may be related to the overall application and transformation of AI in the field of cancer. It is in the initial stage and the effect of promotion and application is limited (88).

Comparing the application in esophageal cancer with other fields, modern research on the prognosis, survival and risk factors for esophageal cancer is bound to become a hot spot in the future. In particular, the word “database” appeared for the first time in 2018. Obviously, with the emergence of big data, the processing and application of large amounts of data has become an important research method. Through big data, we can apply artificial intelligence to conduct comprehensive analysis and extensive research on clinical esophageal cancer. However, at the same time, data require many human and financial resources, making data collection very difficult and valuable, which may be one of the reasons for the lack of cooperation in most studies.

Looking at the entire study, the application of artificial intelligence in esophageal cancer has gone through two significant stages. The early emphasis was on esophageal oncogenes such as p53, classification/identification, and comparison of esophageal cancer. Meanwhile, the risk factors and prognosis of esophageal cancer were intermingled. Recently, the database was coupled with deep learning, convolutional neural networks, and machine learning. These areas are considered is hotspots which are the recent frontier in the examination, diagnosis, and therapy combination choices for esophageal cancer. The subject of artificial intelligence research for esophageal cancer is now approaching a new stage that will lead to the term “precision”. As a result, it will undoubtedly influence preoperative and postoperative nursing and clinical procedures for patients with esophageal cancer.

AI currently appears to have indisputable potential, and in laboratory settings it has shown good enough performance and high enough precision to enhance the care of cancer patients and impact the cancer field more broadly. With the further development of artificial intelligence, the overall development of esophageal cancer toward precise inspection, diagnosis and treatment appears promising. The challenges of applying AI to esophageal cancer in the future may mainly lie in individualized data collection of esophageal cancer (such as information other than clinical indicators, such as genetic information), data quality (such as ethnic differences in data differences), and data processing specifications (electronic health record structure). Inconsistency), AI code reproduction (it is not possible to share code now, it is difficult to reproduce and promote existing results), and auxiliary diagnosis credibility decision-making (results can only be truly credible after being tested in practice.

## Limitation

The study still has certain limitations. Since it takes an article a certain amount of time to reach a certain number of citations, high-quality articles from the last few years have not reached an ideal number of citations, which can cause research deviation. This delay, may also because a delay in the investigation of new scientific frontier. Nonetheless, we added a new metric “Almetric Attention scores” to minimize this limitation. Altmetrics continues to face the issue of not being able to include the continuously expanding media channels in a timely way (e.g., TikTok). Finally, in terms of retrieval time, it may result in the loss of research hotspots in 2022. Only records before April were included this year. Last but not least, our study in WoSCC only contains English literature, which may result in the absence of essential literature in other languages. In addition, future research, databases such as Scopus and Google Scholar might be incorporated and compared for more thorough results.

## Conclusion

In conclusion, artificial intelligence is steadily taking over esophageal cancer research. Although China has the most published articles in this discipline, the United States, the Netherlands, and the United Kingdom have a greater influence and involvement in this field. The frequency of national research collaboration must be increased, particularly for emerging nations. Nations should work hard to retain strong ties with industrialized countries such as the United States. The use of AI in the field of esophageal cancer is generally behind, and the focus of this area will shift to increasing diagnosis accuracy *via* deep learning technology, therapy and prognosis prediction based on big data. The difficulties of AI application in esophageal cancer may be mostly found in personalized data collection, data quality, data processing requirements, AI code reproduction, and helped diagnosis decision-making dependability.

## Data availability statement

The raw data supporting the conclusions of this article will be made available by the authors, without undue reservation.

## Author contributions

The study was created by LW and X-QZ. J-XT gathered the data and prepared the paper. The data were examined by X-TL, H-QY, S-LY, L-FD, and R-LZ. The manuscript was revised and reviewed by LW, X-QZ, and J-XT. The essay was written by all of the writers, and the final version was approved by all of them.

## Funding

This study was supported by the Economic Evaluation Project of Early Diagnosis and Early Treatment of Urban Cancer in Jiangxi Province (No. JXTC2020040486), the National Natural Science Foundation of China (Grant Nos.81960611, and 81960620), and the Nanchang University Students’ innovation and entrepreneurship training program (2022).

## Acknowledgments

The authors would like to express their gratitude to Dr. Jie KUANG and Dr. Yong LIU for their assistance in polishing the English content of this publication.

## Conflict of interest

The authors declare that the research was conducted in the absence of any commercial or financial relationships that could be construed as a potential conflict of interest.

## Publisher’s note

All claims expressed in this article are solely those of the authors and do not necessarily represent those of their affiliated organizations, or those of the publisher, the editors and the reviewers. Any product that may be evaluated in this article, or claim that may be made by its manufacturer, is not guaranteed or endorsed by the publisher.
