# Supplementary material for: Global research trends of artificial intelligence applied in esophageal carcinoma: A bibliometric analysis (2000-2022) via CiteSpace and VOSviewer
Source: Front Oncol. 2022 Aug 25;12:972357. doi: 10.3389/fonc.2022.972357 (PMC9453500; doi:10.3389/fonc.2022.972357)
Supplement: Supplementary file 1 [file DataSheet_1.docx]

| **Rank** | **Country** | **Counts** | **H-index** | **TLS** | **Total citations** | **Average Publication Year** |
| --- | --- | --- | --- | --- | --- | --- |
| 1 | China | 317 | 29 | 164 | 4255 | 2018.68 |
| 2 | United States | 232 | 45 | 253 | 9927 | 2015.84 |
| 3 | United Kingdom | 123 | 24 | 74 | 1448 | 2015.62 |
| 4 | Japan | 90 | 25 | 86 | 3127 | 2013.86 |
| 5 | Germany | 86 | 20 | 175 | 2775 | 2015.30 |
| 6 | Netherlands | 84 | 25 | 184 | 2828 | 2016.33 |
| 7 | Canada | 43 | 15 | 80 | 1562 | 2015.64 |
| 8 | Italy | 43 | 9 | 119 | 1085 | 2017.93 |
| 9 | Australia | 37 | 12 | 87 | 1880 | 2014.59 |
| 10 | France | 34 | 12 | 82 | 1665 | 2016.97 |

**Table S1** Top 10 productive countries/regions related to AI on EC

**Table S2** Subjects of key words’ cluster analysis (2000-2022)

| **Cluster number** | **Silhouette** | **Size** | **Log-likelihood (LLR)** |
| --- | --- | --- | --- |
| 0 | 0.801 | 56 | radiotherapy; pet; computed tomography; radiomics; texture analysis; |
| 1 | 0.921 | 47 | barrettes-esophagus; cancer; apoptotic index; esophageal squamous cell carcinoma; pattern; |
| 2 | 0.927 | 46 | microscopy; high grade dysplasia; surveillance; optical coherence tomography; intestinal metaplasia; |
| 3 | 0.944 | 44 | esophageal cancer; artificial neural network; gene expression profiling; immune infiltration; endoscopy; |
| 4 | 0.805 | 42 | outcome; system; multicenter; cancer; association; |
| 5 | 0.874 | 41 | deep learning; artificial intelligence; gastrointestinal endoscopy; convolutional neural network; image segmentation; |
| 6 | 0.89 | 39 | human microrna; database; expression; integrome network; comparative proteomics; |
| 7 | 0.807 | 33 | pik3ca gene; growth factor receptor; cell lung cancer; positron emission tomography; esophageal; |
| 8 | 0.877 | 32 | risk factor; gastroesophageal reflux; hepatocellular carcinoma; cancer incidence; systematic analysis; |
| 9 | 0.883 | 29 | breast cancer; prostate cancer; p53; apoptosis; mutational signature; |
| 10 | 0.932 | 27 | squamous cell carcinoma; adenocarcinoma; cdna; comparative genomic hybridization; lymph node metastase; |
| 11 | 0.931 | 27 | colorectal cancer; gastric cancer; screening; cancer risk; |
| 12 | 0.895 | 22 | bioinformatics analysis; esophageal neoplasms; potential gene targets; esophageal carcinoma (esca); machine learning; |
| 13 | 0.929 | 20 | microrna; association prediction; adaptive radiotherapy; disease; tumor; |
| 14 | 0.994 | 17 | health care; cost effectiveness; society; quality of life; management |
| 15 | 0.952 | 15 | artificial intelligence; computer-assisted diagnosis; deep learning; gastroscopy; gastroenterology; |
| 16 | 0.969 | 11 | training; high volume; centralization; early gastric cancer; learning curve; |
| 17 | 1 | 10 | prognostic potential; interpretive transforms machine vision; diagnostic histopathology; nuclear and lesion signatures; gastrointestinal endoscopy; |
| 18 | 0.989 | 6 | treatment response; radiomics analysis; predictor; oesophageal neoplasms; symptom |
| 20 | 0.993 | 5 | carotid endarterectomy; percutaneous coronary intervention); surgical volume; acute myocardial infarction; abdominal aortic aneurysm; |
| 21 | 0.995 | 5 | industry sector; attributable fraction; carcinogen; cancer burden; occupation; |
